# Unrecognized geriatric depression in the emergency Department of a Teaching Hospital in Nepal: prevalence, contributing factors, and metric properties of 5 item geriatric depression scale in this population

**DOI:** 10.1186/s12888-020-02910-8

**Published:** 2020-11-11

**Authors:** Roshana Shrestha, Anmol Purna Shrestha, Abha Shrestha, Barbara Kamholz

**Affiliations:** 1 Department of General Practice and Emergency Medicine, Kathmandu University School of Medical Sciences, Dhulikhel, Nepal; 2 Department of Community Medicine, Kathmandu University School of Medical Sciences, Dhulikhel, Nepal; 3 Volunteer Clinical Professor of Psychiatry, University of California at San Francisco, San Francisco, CA USA

**Keywords:** Depression, Elderly, Emergency department, Geriatric depression scale, LMICs, Heart and mind disease, Illiteracy, Nepal, Pain

## Abstract

**Background:**

Depression is prevalent but poorly recognized in the Emergency Department (ED). We aimed to identify the frequency of unrecognized geriatric depression and its possible determinants in the ED using the 15-item geriatric depression scale (GDS). We also aimed to explore the performance of the shorter, five-item GDS as compared to the 15-item GDS.

**Methods:**

This was a cross-sectional study of the ED patients ≥ 60 years. The previously validated Nepali version of GDS-15 screened the sample into “no”, “mild-moderate” and “severe” depression using cutoff values of 4/5, and 8/9 respectively. Demographic and socioeconomic factors, comorbidities and health seeking behaviors were studied and the relation was assessed with binary (Chi-square and Kruskal-Wallis test) and multinomial regression analysis. The performance of GDS-5 was compared with the GDS-15 as standard. Cronbach’s alpha was calculated to investigate reliability. Validity was assessed by calculating sensitivity, specificity, positive predictive value, negative predictive value, Spearman’s correlation, receiver operating characteristic curve, and kappa coefficient.

**Results:**

Two hundred eighty patients were enrolled with an overall prevalence of unrecognized depression of 45.7% [104 (81.3%) mild-moderate depression, and 24 (18.8%) severe depression]. The mean age of the sample was 71.36 with female predominance (61%), and 82.5% were illiterate. In the binary analysis, those who had more pain, visited the ED more often, had musculoskeletal diseases and sleep problems, mobility problems, visited local healers previously, and who reported self-perceived “heart and mind” disease showed statistically significant differences among the three categories. In multinomial regression analysis, visits to local healers, sleep problems and frequency of pain were significantly related to depression. The sensitivity, specificity, area under curve and Spearman’s correlation of GDS-5 were 75.8%, 96%, 0.919, and 0.827 respectively. Cronbach’s alpha for GDS-5 was low (0.416), therefore a new version was proposed which improved the sensitivity to 90.6% and Cronbach’s alpha to 0.623.

**Conclusions:**

Unrecognized geriatric depression was highly prevalent which urges the need for ED-based interventions for screening and referral. The proposed brief GDS-5 correlated well with the GDS-15 with better validity and internal reliability and offers a more expeditious form of screening for geriatric depression in emergency settings in Nepal.

## Background

The World Health Organization (WHO) estimates that the number of aged population (age ≥ 60 years) will reach two billion by 2050, 80% of whom will be living in low- and middle-income countries (LMIC) [1]. In the WHO fact sheet, depression is listed as one of the several common health conditions associated with ageing which often coexists with other several conditions like hearing loss, cataract and refractive errors, osteoarthritis, chronic obstructive pulmonary disease (COPD), diabetes mellitus (DM), and dementia. In 2017, depressive disorders were the third leading cause of “years lived with disability” (YLD) and are expected to become the leading cause of disease burden by 2030 [2]. Depression is costly and carries significant comorbidity. Loss of productivity and role performance due to depression are profound [3]. Although demographic changes point to significant increases elderly populations, the study of the prevalence, impact, and options for treatment of depression among the elderly in LMICs is in its infancy. The reported “treatment gap” in depression is 90% in Nepal [4]. Given the limited resources for both mental and geriatric healthcare, depression in increasing numbers of elderly poses a significant risk of socioeconomic and medical burden for LMICs [5].

Nepal is one of the world’s poorest countries, and considered a fragile state by the World Bank [6]. The WHO Study on Global AGEing and Adult Health (SAGE) identified depression in LMICs to be 4.7% [7]. However, in Nepal, from 17.3 to 89.1% of elders in elder care homes, 25.5 to 60.6% of elders living in the community, and 53.2 to 57.1% of elders in hospitals suffer from depression [8]. Manandhar et al. found an adjusted prevalence of depression among elders of 53.1% in the Kavre District, a largely rural area and the site of our study [9]. Impoverishment contributes significantly to difficulty in accessing care in rural areas of Nepal such as Kavre district, with geographical challenges and a high illiteracy rate. Yet only 0.17% of Nepal’s annual budget is available for mental health issues, and WHO reports just 18 psychiatric outpatient facilities in the country in 2006 [10]. As of 2016, there were only 50 psychiatrists in Nepal, many of whom work in urban areas [11]. Depression is a stigmatized condition (heart and mind disease) that limits elders, and their families, from bringing it to the attention of healthcare providers [12].

Geriatric patients visiting the Emergency Department (ED) have diverse patterns of service use [13]. In comparison to younger patients, geriatric patients have a higher rate of ED visits and admissions with a greater level of urgency. Their ED stay is longer with repeated visits and experience higher rates of adverse health outcomes after discharge. ED visit frequency has a significant relationship with depressive symptoms [14] and these patients are more likely to return to the ED [15]. Geriatric depression is known to cause significant distress and disability, which exacerbates, and is often exacerbated by, existing medical conditions [16]. Despite being common in older ED patients, depression often goes unrecognized by emergency physicians (EP) as it is masked by other presenting complaints and is thus unlikely to receive mental health referrals [17–19]. Geriatric patients presenting to Dhulikhel Hospital constitute approximately 25% of all presentations. With the rapid growth of the older adult population, it is critical that EP, the frontliners, be prepared for the evaluation and management of mental health disorders including depression in the ED. In Nepal, no survey has been performed for prevalence of depression among geriatric patients presenting to the ED. Appropriate brief and validated screening tools and training of the ED staff would improve the recognition of geriatric depression in a busy ED like ours, leading to appropriate referral for further management [20–23]. The Geriatric Depression Scale (GDS) is a widely used screening tool to identify depression risk in geriatric patients. The GDS was originally a 30-item questionnaire (GDS-30) [24], and it has been validated in a large sample of geriatric patients. A 15-item form (GDS-15) has been validated and is now widely used [25], as well as a 5-item version (GDS-5), which has been shown to be as sensitive in detecting depression in multiple clinical settings (hospital, outpatient clinic, and nursing home) [26].

The first aim of this study was to identify the prevalence of previously unrecognized depression and its possible determinants in geriatric patients in the ED using the previously validated 15-item Nepali version of GDS. The detailed nature of GDS-15 is time consuming and hence may not feasible to be used as a quick depression screening tool in ED set-up. Since we found no studies using the GDS-5 in Nepal in the ED setting, we also aimed to explore the performance of the shorter five item GDS that could be more efficient to be used in a busy ED in future practice.

## Methods

### Study design/setting

This was a cross sectional, prospective, quantitative, observational study over a period of 7 months from July 15, 2019-Feb 14, 2020 at the ED of Dhulikhel Hospital-Kathmandu University Hospital with approximately 20,000 visits annually.

### Participants

The study was approved by the Institutional Review Committee of Kathmandu University School of Medical Sciences. Convenience samples of ED patients aged 60 and over were included consistent with WHO definition [1]. Patients who were stable enough and eligible to participate as per the assessment by the treating physician were included. Those who had Glasgow coma scale (GCS) < 15, refused to participate, had difficulty with communication, had previous diagnosis of depression/dementia or who were under current treatment with a psychiatrist for depression were excluded. All eligible patients were approached during the office hours (0900–1600) of weekdays as per availability of the research assistant in the ED. The questionnaire was administered verbally by the authors or trained research assistant in less crowded area in ED. The patients were interviewed once being cleared from urgent care needs by the treating EP. They were informed about the purpose and procedures of the study and the right to withdraw from the study. Written consent was obtained from patients who could read and write, while fingerprints were collected from those who were unable to do so. The assistant took assent from a family member regarding participation whenever necessary. Participants who screened positive for depression with the GDS-15 were counseled to attend psychiatry clinic at the hospital. Further follow up of the patient was not done during this phase of the study.

### Variables and data measurement

The research assistant obtained and recorded the patient’s demographic data (age, gender, religion, marital status, educational level, present occupation, family support), mobility status, alcohol consumption, presence of pain, pattern of sleep, medical history, and previous visits to local healers and to the ED during the prior year. The past medical history was reconfirmed by checking the Electronic Medical Record of the ED. Each patient was asked about their self-perception of the family income and presence of the “heart and mind disease” (“*man ko rog*”) which is the closest idiom for depression in Nepali. We assumed that the patients would not understand the terms “depression” or “*avasad,*” as illiteracy among the elders in Nepal is very high. Psychiatric illnesses or “disease of brain or mind” is one of the most stigmatized problem in Nepali culture. The phrases “psychiatric illness” and “disease of brain or mind” would cause stigmatization and there was a concern that the patients would avoid interactions; therefore, these terms were avoided.

We used the GDS-15, which was derived from the original GDS-30 with the highest correlation with depressive symptoms. The GDS-30 is one of the most widespread and reliable scales for the evaluation of depression among the elderly. It was first created by Brink [24] in response to the need for a diagnostic instrument, specifically for the elderly, that could distinguish a patient suffering from depressive illness from other pathologies such as cognitive impairment. The GDS-15 has been shown to perform similarly to the original scale, with a sensitivity of 86.5% and sensitivity of 82.7% (Youden’s index = 0.692), using a cutoff score of 4/5 for the detection of depression [27, 28]. The GDS-15 has a Cronbach’s alpha of 0.82 and the correlation between the GDS-30 and the GDS-15 is 0.84. Each item covers the individual’s experiences during the last week and somatic nonspecific items like fatigue and concentration are avoided. Of the 15 items, 10 items if answered positively (Yes) and the remaining 5 items if answered negatively (No) indicate the presence of depression. The GDS is useful in the ED given that it has simple “yes or no” responses. A prior study by Yesavage and Sheikh (1986) has suggested that a score of 0–4 indicates no depression, 5–8 indicates mild depression and 9–15 severe depression [25]. The scale is validated internationally [29] and has been adequately translated and validated in Nepal [30, 31] as well (Additional file 1). Permission was obtained from the primary authors for its use for this study. The GDS-5 has been found to be as effective as the 15-item scale, with 97% sensitivity and 85% specificity [32]. An additional study found that the GDS-5 with a cutoff score of 2/3 was as effective as GDS-15 for depression screening. There was a marked reduction in administration time, which may be essential in a busy ED [33]. In our study, the performance of GDS-5 was assessed for its potential regular use in ED in our context.

### Sample size

**Fig. 1:**
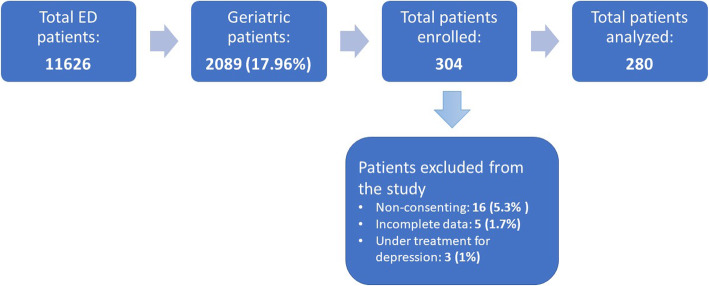
Flow chart of study sample

To our knowledge, no other studies have been conducted in Nepal regarding geriatric depression in ED settings. We searched various studies done in relation to geriatric depression in the ED in our part of world. In the study done in an ED in India, psychiatric diagnoses in the study sample included dementia (9.5%), depressive disorders (8.2%), adjustment disorder (3%), and anxiety (not otherwise specified) disorder in 3.4% of participants [34]. The low prevalence of depression in this study may have been due to use of more specific screening tool. Among the recent studies, the highest prevalence found was 22.72% in Australia in 2013 which also used GDS-15 as the screening tool [35]. These figures formed the basis for assessment of our sample size. We calculated the sample size for our study using this reference as 270. The details of flow of the study sample is depicted in Fig. 1.

### Data analysis/statistical methods

We entered the data in Microsoft Excel and analyzed the data with SPSS version 22. Analysis primarily involved descriptive statistics (proportion, mean, median, IQR, range). We categorized the patients using the GDS-15 scale into three groups; no depression, mild depression and severe depression with the cut off values of 4/5 and 8/9 respectively. Other demographic variables were dichotomized, as: educational status (no formal education vs any formal education), occupation (employed vs unemployed) and marital status (married vs single at present due to any cause). Categorical variables were compared with the chi square test. The Independent-samples Kruskal-Wallis test was used to compare the continuous variables with the three categorical variables. Multinomial regression analysis was applied to find specific factors associated with geriatric depression. The factors with *P*-value < 0.2 in former bivariate analysis were included for the analysis. Statistically, when *P*-value are < 0.05, the independent variables are likely to have impact on the dependent variable, depression.

A Kappa value between the research assistant and one of the authors (RS, APS) was performed to assess inter-rater reliability in 20 randomly selected cases. The Kappa result is interpreted as follows: values ≤0 as indicating no agreement and 0.01–0.20 as none to slight, 0.21–0.40 as fair, 0.41–0.60 as moderate, 0.61–0.80 as substantial, and 0.81–1.00 as almost perfect agreement [36].

The validity of the five-question depression screen was compared to the standard 15 question depression screen by calculating sensitivity, specificity, positive predictive value and negative predictive values using a cutoff score of 2/3, and were reported as proportions using 95% confidence intervals (95% CIs) [37]. Sensitivity or true positive rate is referred to the proportion of patients who scored positive for depression on the GDS-5 and GDS-15. Specificity or true negative rate is the proportion who had scored negative for depression with the GDS-5 and GDS-15. Positive predictive value is the proportion of true positives among all positives and negative predictive value is proportion of true negatives among all negatives. The construct validity was measured by using the Spearman’s correlation test that compares GDS-5 with GDS-15. The Spearman correlation coefficient, r_s_ of + 1 indicates a perfect association of ranks, a r_s_ of zero indicates no association between ranks and a r_s_ of − 1 indicates a perfect negative association of ranks [38]. In order to study the discriminant validity, the receiving operating characteristic (ROC) curve was created, and the area under the curve (AUC) was calculated. The closer the AUC value is to 1.0, the greater the instrument’s ability to differentiate between depressed and nondepressed patients. An AUC higher than 0.75 confers to the tool a moderate discriminative validity; while an excellent one is demonstrated by a value ≥0.90.

The agreement between the GDS-15 and the GDS-5 was also calculated with a kappa coefficient. A reliability analysis was carried out on the GDS-15 scale items and the GD − 5 scale. The reliability of the GDS score was assessed in terms of internal consistency by calculating Cronbach’s alpha. Alpha values closest to 1 are considered best: excellent if > 0.9, good if > 0.8, and acceptable if > 0.7 [39].

## Results

One hundred and twenty-eight patients (45.7, 95% CI: 39.8–51.7) positively screened for depression among the 280 geriatric ED patients with GDS-15 using a cut off value of 4/5. The prevalence of previously unrecognized depression was 42.2% (95% CI: 32.8–52) in males and 48% (95% CI: 40.3–55.7) in females, but the difference was not statistically significant (*P-*value = 0.35). The mean GDS-15 score was 4.43 (95% CI: 4.11–4.78, SD ±2.87, range 0–13). Among all the positively screened patients (*n* = 128), 104 (81.3, 95% CI: 73.4–87.6) screened positive for mild depression (score 5–8) and 24 (18.8, 95% CI: 12.4–26.6) were screened positive for severe depression (score > 8) (Table 1). When GDS-5 was used as the screening tool with a cutoff score of 2/3, 103 (36.8, 95% CI: 31.1–42.7) screened positive for depression with mean score of 1.3 (95% CI: 1.16–1.43, SD ±1.146, Range 0–5) (Table 2). There was a high degree of agreement between one of the authors (RS, APS) and the research assistant on 20 randomly selected geriatric patients (Cohen’s Kappa k = .841(95% CI: .633–1.05), *P-*value<.001).

**Table 1:** Prevalence of geriatric depression according to GDS-15 (*n* = 280)

Category	GDS-15 score	*n* (%)	Subtotal *n* (%)
Negatively screened for depression	0	12 (4.3)	152 (54.3)
	1	38 (13.6)	
	2	38 (13.6)	
	3	31 (11.1)	
	4	33 (11.8)	
Positively screened for mild depression	5	26 (9.3)	104 (37.1)
	6	35 (12.5)	
	7	21 (7.5)	
	8	22 (7.9)	
Positively screened for severe depression	9	11 (3.9)	24 (8.6)
	10	5 (1.8)	
	11	4 (1.4)	
	12	2 (.7)	
	13	2 (.7)

**Table 2:** Prevalence of geriatric depression according to GDS-5 (*n* = 280)

Category	GDS-5 score	*n* (%)	Subtotal *n* (%)
Negatively screened for depression	0	78 (27.9)	177 (63.2)
	1	99 (35.4)	
Positively screened for depression	2	60 (21.4)	103 (36.8)
	3	31 (11.1)	
	4	9 (3.2)	
	5	3 (1.1)	

### Associations of geriatric depression

Mean age of the total sample was 71.36 years (95% CI: 70.42–72.31, SD ± 8.06, range: 60–94) with no significant difference by gender. The majority of patients were females (61%) largely single (78.9%) (*P-*value < .001). Among them, 82.5% had no formal education and females were more illiterate (94% vs 64%, *P-*value < .001). Male patients consumed significantly more alcohol than their female counterpart (24% vs 14%, *P-*value = 0.036). Two hundred and eight (74.3%) patients had previous visits to ED in the last 1 year, and the proportion was higher in females (78%) in comparison to males (68%) (*P-*value = 0.051). Comorbidities like COPD, cardiovascular disease (CVD), musculoskeletal (MSK) disease and DM were present in 59.3, 37.1, 16.8 and 7.9% of the study population respectively without significant difference by gender. Only one patient among the study group had no family support.

**Fig. 2:**
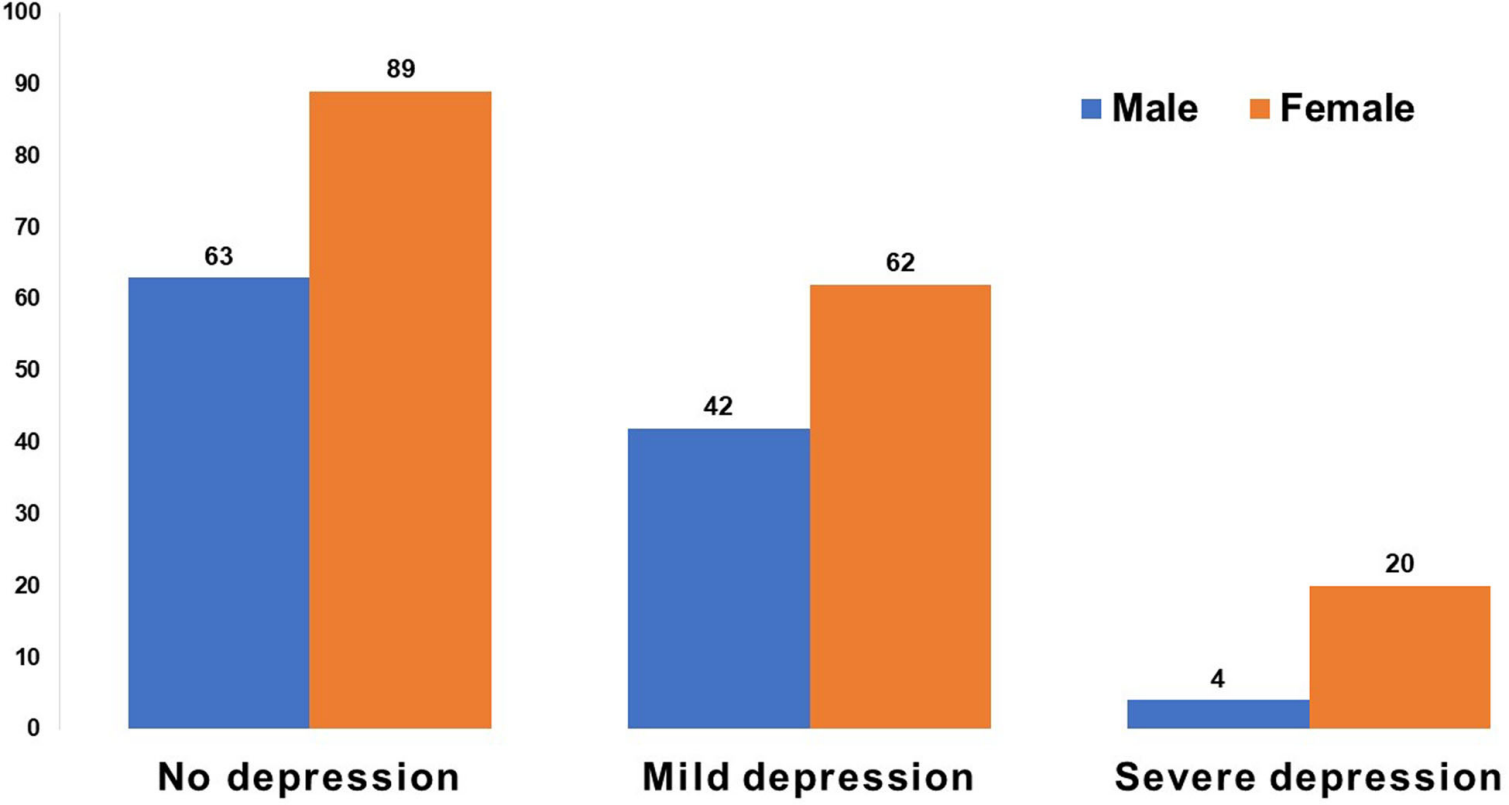
Categories of geriatric patients according to the GDS-15

The association of the demographic data, comorbidities and health seeking behavior with depression was evaluated (Table 3). The negative, mild depression and severe depression groups were similar with regard to age, gender, marital status, educational level, employment, financial condition, presence of pain, presence of comorbid conditions and previous ED visits. Females consisted 83% of those who were severely depressed, however the difference was not statistically significant (*P-*value = 0.064) (Fig. 2). Those who self-reported having the “heart and mind disease” had higher GDS-15 scores (*P-*value < .001). Furthermore, the depressed and severely depressed subjects, compared with non-depressed subjects, had more disturbed sleep (*P-*value < .001) and visited the local healers more frequently(*P-*value < .001). Similarly, those who had more pain, visited the ED more often and who had MSK diseases showed statistically significant differences among the three categories of depressive status (*P-*value = 0.014, 0.047 and 0.016 respectively).

**Table 3:** Association of socio-demographic characteristics, comorbidities and health seeking behavior with depression categories (*n* = 280)

Variables	Total (*n* = 280)	GDS-15 score < 5 (*n* = 152)	GDS-15 score 5–8 (*n* = 104)	GDS-15 score 9–15 (*n* = 24)	*P-* value
GDS-15 Score, Median (IQR)	4 (2–6)	2 (1–3)	6 (5.5–7)	10 (9–11)	NA
GDS-5 Score, Median (IQR)	1 (0–2)	0.5 (0–1)	2 (1–2)	3 (3–4)	**<.001**^**a**^
Age, median (IQR)	70 (65–70)	70 (65–76)	71 (65–78)	68 (64.5–75)	0.458^a^
Female, n (%)	171 (61)	89 (58.6)	62 (59.6)	20 (83.3)	0.064^b^
Marital status-single, n (%)	109 (38.9)	59 (38.8)	43 (41.3)	7 (29.2)	0.544^b^
No formal education, n (%)	231 (82.5)	126 (82.9)	84 (80.8)	21 (87.5)	0.723^b^
unemployed, n (%)	195 (69.6)	101 (66.4)	79 (76)	15 (62.5)	0.194^b^
Finance resource (not enough and just enough), n (%)	36 (12.9)	19 (12.5)	11 (10.6)	6 (25)	0.161^b^
Alcohol, n (%)	50 (17.9)	25 (16.4)	19 (18.3)	6 (25)	0.591^b^
Pain, n (%)	217 (77.5)	113 (74.3)	83 (79.8)	21 (87.5)	0.278^b^
Frequency of pain in 1 week, median (IQR)	3 (2–4)	3 (0–4)	3 (2–4)	4 (2.5–5.5)	**0.014**^**a**^
Disturbed sleep, n (%)	159 (56.8)	68 (44.7)	76 (73.1)	15 (62.5)	**<.001**^**b**^
Visit to local healer, n (%)	85 (30.4)	21 (13.8)	52 (50)	12 (50)	**<.001**^**b**^
Self-perceived presence of heart and mind disease, n(%)	94 (33.6)	30 (19.7)	52 (50)	12 (50)	**<.001**^**b**^
Mobility, needs support, n (%)	89 (31.8)	43 (28.3)	42 (40.4)	4 (16.7)	**0.031**^**b**^
Previous ER visit, n (%)	208 (74.3)	109 (71.5)	82 (78.8)	17 (70.8)	0.405^b^
Frequency of ED visits in last 1 year, median (IQR)	2 (0–3)	1.5 (0–3)	2 (1–3)	3 (0–5)	**0.047**^**a**^
COPD, n (%)	166 (59.3)	88 (57.9)	62 (59.6)	16 (66.7)	0.716^b^
CVD, n (%)	104 (37.1)	60 (39.5)	36 (34.6)	8 (33.3)	0.675^b^
DM, n (%)	22 (7.9)	14 (9.2)	6 (5.8)	2 (8.3)	0.601^b^
MSK, n (%)	47 (16.8)	24 (15.8)	14 (13.5)	9 (37.5)	**0.016**^b^

^a^Independent samples Kruskal Wallis test, ^b^Chi square test

Table 4 illustrates the estimates of multinomial logistic regression coefficients, their *p* value and odds ratios for each variable after controlling for confounders. Those variables which showed *P-*value of < 0.2 in the bivariate analysis of non-depressed vs mild and severe depression (Table 3) (gender, occupation, financial resource, mobility, sleep, frequency of pain, frequency of ED visit, self perceived “heart and mind disease”, visit to the local healer and presence of MSK disease) were chosen for the multinomial logistic regression analysis. The “no depression” category was taken as reference. The Hosmer-Lemeshow goodness-of-fit test indicated that the model described the data well (*P-*value = 0.305). Visits to local healers were associated with both mild depression and severe depression, independently of other factors (*P-*value = 0.003 and 0.019 respectively). Those who reported to have disturbed sleep were 2.3 times more likely to screen positive for mild depression as opposed to no depression (*P-*value = 0.006). Patients who experienced more frequent pain were 1.3 times more likely to screen positive if they were severely depressed versus people with no depression (*P-*value = 0.05). No other variable was associated with either mild or severe depression in multinomial regression analysis.

**Table 4:** Multinomial logistic regression analysis of the relationship between different variables and different severity of depression as compared to not depressed. (*n* = 280)

Variables	Mild depression vs no depression OR (95% CI)	*P-*value	Severe depression vs no depression OR (95% CI)	*P-*value
**Gender**				
Female	1	0.756	1	0.087
Male	1.09 (.617–1.943)		0.358 (.110–1.161)	
**Occupation**				
Not employed	1	0.149	1	0.768
Employed	0.606 (.306–1.197)		0.854 (.299–2.437)	
**Disability**				
Needs support	1	0.699	1	0.183
On their own	1.13 (.608–2.099)		0.426 (.121–1.496)	
**Self-perceived financial resource**				
Not enough	1	0.853	1	0.248
enough	1.091 (.432–2.757)		.493 (.149–1.635)	
**Self-perceived mind and heart disease**				
Absent	1	0.295	1	0.217
Present	2.455 (.458–13.170)		5.316 (.375–75.301)	
**Disturbed sleep**				
No	1	**0.006**	1	0.504
Yes	2.326 (1.275–4.244)		1.411 (.514–3.877)	
**Visit to local healer**				
Yes	1	**0.003**	1	**0.019**
no	0.072 (2.326–1.275)		0.04 (.003–.595)	
**MSK disease**				
Present	1	0.594	1	0.609
Absent	1.266 (.532–3.009)		.734 (.224–2.402)	
**Frequency of pain during last week**	1.019 (.864–1.201)	0.827	1.318 (1–1.737)	**0.05**
**Frequency of ED visits in last year**	1.062 (.948–1.191)	0.3	1.111 (.942–1.310)	0.212

Significant *P-*values are bolded

### Performance of GDS-5 vs GDS-15 in our sample

The sensitivity of the brief original GDS-5 (items 1,4,8,9,12 from GDS-15) when compared to the standard GDS-15 was 75.78% (97/128; 95% CI: 67.42–82.91), with a specificity of 96.05% (146/152; 95% CI: 91.61–98.54) using a cutoff score of 2/3 (Youden index = 0.718). Significant agreement was found between the 15-item GDS and the 5-item GDS (kappa = 0.73). A reliability analysis was carried out on the GDS-15 scale items and the GDS-5 scale. The Cronbach’s alpha score reached acceptable reliability (alpha = 0.714) for the GDS-15, however it was low for the GDS-5 (alpha = 0.416). As illustrated in Table 5, items 1, 3, 4, 7 and 14 in the GDS-15 appeared to be the worthiest of retention, resulting in a “decrease” in the alpha if deleted. The items 9, 11, 13 and 15 would “increase” the alpha if deleted. Therefore, a new version of the GDS-5 for our population was proposed selecting these 5 items from the GDS-15 with the highest corrected item- total correlation (items 1, 3, 4, 7 and 14).

**Table 5:** Item-Total Statistics of GDS-15

Items in GDS15	Scale Mean if Item Deleted	Scale Variance if Item Deleted	Corrected Item-Total Correlation	Cronbach’s Alpha if Item Deleted
Item 1^a^	4.2652	7.239	.427	.689
Item 2	4.2545	7.478	.292	.703
Item 3^a^	4.1004	6.889	.453	.682
Item 4^a^	3.9283	6.973	.381	.692
Item 5	4.2867	7.421	.359	.696
Item 6	4.0215	7.000	.380	.692
Item 7^a^	4.1685	7.112	.394	.691
Item 8	4.3011	7.449	.366	.696
Item 9^b^	4.2222	7.735	.147	.718
Item 10	3.8674	7.274	.265	.707
Item 11^b^	4.3978	8.053	.146	.714
Item 12	4.1470	7.183	.349	.696
Item 13^b^	3.8387	7.481	.189	.717
Item 14^a^	4.0430	6.905	.425	.686
Item 15^b^	4.1792	7.565	.200	.713

^a^Items of GDS-15 resulting in a “decrease” in the alpha if deleted (items for new GDS-5),

^b^Items of GDS-15 resulting in an “increase” in the alpha if deleted

Sensitivity, specificity, diagnostic accuracy, and positive and negative predictive values were calculated for the proposed 5-item scale and compared with the original one (Tables 6 and 7). The validity was recalculated which improved the sensitivity to 90.62 (95% CI: 84.20 to 95.06%) (Youden index = 0.755) and the Cronbach’s alpha increased to 0.623 from 0.416. Thus, the detection rate of geriatric depression with the new scale was higher (139 vs 103) (Table 6). The agreement between the 15-item GDS and the proposed 5-item GDS was also higher than with the original one (kappa = 0.75 vs 0.73) (Table 7). Other measures for validity like Spearman’s correlations (strong correlation for both) and AUC (excellent discriminant validity for both) are depicted in Table 7.

**Table 6:** Assessment of original and proposed GDS-5 accuracy in relation to the GDS-15

	GDS-15 n (%)	
	Depressed (*n* = 128)	Not depressed (*n* = 152)
**Original GDS-5**		
Depressed (*n* = 103)	97 (75.8)	6 (3.9)
Not depressed (*n* = 177)	31 (24.2)	146 (96.1)
**Proposed GDS-5**		
Depressed (*n* = 139)	116 (90.6)	23 (15.1)
Not depressed (*n* = 141)	12 (9.4)	129 (84.9)

**Table 7:** Performance of original and proposed GDS-5 against GDS-15

	Original GDS-5	Proposed GDS-5
Sensitivity, % (95% CI)	75.8 (67.42–82.91)	90.6 (84.2–95.06)
Specificity, % (95%CI)	96 (91.61–98.54)	84.9 (78.17–90.16)
Positive likelihood ratio, (95% CI)	19.2 (8.71–42.31)	5.99 (4.09–8.76)
Negative likelihood ratio, (95%CI)	0.25 (0.19–0.34)	0.11 (0.06–0.19)
Positive predictive value, % (95%CI)	94.2 (88.00–97.27)	83.45 (77.50–88.06)
Negative predictive value, % (95% CI)	82.5 (77.59–86.51)	91.49 (86.21–94.87)
Accuracy, % (95% CI)	86.8 (82.25–90.52)	87.5 (83.05–91.14)
AUC (area under curve) (95% CI)	0.919 (0.887–0.951)	0.929 (0.900–0.959)
Cronbach’s Alpha	0.416	0.623
kappa	0.73	0.75
Spearman’s correlation coefficient, r_s_	0.827	0.860

## Discussion

Depression is a serious mental health problem in geriatric patients. To our knowledge, this is the first ED based survey in Nepal to explore the burden of geriatric depression. In this study, screening for depression with the GDS-15 among the ED patients aged ≥60 years revealed a prevalence of 45.7%. Mild-moderate and severe depression was found in 37.1 and 8.6% respectively. This finding closely approximates the prevalence of geriatric depression in the community as per study findings of Manandhar et al. and Yadav et al. (53.1% in Kavre district and 55.8% in Sunsari and Morang districts respectively) [9, 40]. Almost all of the participants in our study had some support at home and were therefore brought to the ED. We might have missed elders who needed medical care but couldn’t access the ED. However, the prevalence we found is higher when compared to the studies done in the ED of high resourced countries [8.2% (India) [34], 16.5% (USA) [23], 17% (USA) [21], 22.75% (Australia) [35], 27% (USA) [19] and 42% (Hong Kong)] [41]. Overall, our study suggests that the ED is an important place for identification of depressed elderly patients in Nepal. Thus, there is an urgent need of intervention to detect this prominent and highly disabling disorder.

### Associations of geriatric depression

We sought to learn what socioeconomic/demographic background, comorbidities and health seeking behaviors of the study cohort were associated with depression, an assessment of which could form the basis for effective interventions. We found no statistical significant associations between depression and age, sex, education, marital status, occupation, alcohol consumption or mobility after multivariate analysis. Increased frequency of pain and ED visits, self-reported “heart and mind” disease and presence of MSK diseases were the independent variables associated with depression during binary analysis. Simkhada et al. [42] found that illiteracy, physical immobility, presence of physical health problems were significantly associated with depression in older adults. In our study, although the prevalence of severe depression was higher among females than for the males, the difference was not found to be statistically significant. Manandhar et al. [9] also found no relation between age and gender of the older patients and depression, however they found significant associated with rural habitation, illiteracy, limited time provided by families, and exposure to verbal and/or physical abuse. In our study, illiteracy had no association with depression [82.5% overall, females were more illiterate than males (94% vs 64%)].

### Depression, comorbidities and health seeking behaviors

Depression is also highly comorbid with common non-communicable diseases, adding significant morbidity and mortality. In our study the comorbidity among the total sample with COPD, CVD, DM and MSK disease was 59.3, 37.1, 16.8 and 7.9% respectively with no significant difference between by gender. Those who had MSK disease showed significant higher incidence of severe depression with binary analysis. However, this difference did not persist in the multivariate logistic regression. The proportion of other comorbidities were similar in depressed and not depressed groups in our study population. Those who reported to have disturbed sleep were 2.3 times more likely to screen positive for mild depression as opposed to no depression with multivariate logistic regression. The relationship between sleep and depression is complex and reciprocal- depression can cause sleep problems and sleep problems may contribute to depressive illness. Therefore, those who report having sleep problems should be screened for depression. Patients who experienced more pain were 1.3 times more likely to screen positive for severe depression. Pain and depression create a vicious cycle. Visits to local healers were associated with both mild depression and severe depression, independently of other factors. The practice of seeking help from local healers is a deeply ingrained cultural norm in Nepal. Persons in remote areas may first seek local traditional healers; they are far more available and less expensive than psychiatrists. They are reluctant to change and believe more in traditional healers than allopathic medicine especially for disease of “heart and mind.” The reason maybe that they trust the local healers more as they are from their own locality with similar culture and tradition. Further in-depth qualitative study is needed to understand this subject. Another noteworthy finding to mention is that the confidence interval for self-perceived mind and heart disease was wide for both categories of depression. The reason may be that the sample size was not enough to describe this variable. Although the patients reported to have the mind and heart disease when asking, no patient presented to the ED with this primary problem. Unfortunately, overall, our study did not identify many specific clues with respect to risk for depression. Almost one third of the sample (74.3%) had previous visits to the ED in the last 1 year. The binary analysis showed that the frequency of ED visits was associated with higher median GDS-15 score. However, the multinomial logistic regression failed to prove the association. Previous surveys have shown that delays in onset of treatment lead to frequent revisits to EDs [15], Another study suggested that a community outreach program and links to social supports could improve the management of geriatric depression [35]. An integrated approach to detection and management of geriatric patients in the ED is essential.

### Metrics of short GDS-5 score

In Nepal, EM is in the preliminary phase and several EM training modules are currently practiced, with different curricula and duration [43]. In a study by Meldon, EP recognized only 13% of depression patients [17]. Unquestionably ED is on the frontline of the mental health crisis, therefore, appropriate screening and intervention ED protocols are needed to target these high-risk elderly patients. It is important that the screening tool be short yet valid and reliable enough to be used in a busy daily practice in the ED. The GDS is a commonly used screening tool for depression among the elderly and previous study has validated it in the Nepalese population [31]. However, the original GDS with 30 and even 15 questions would be too time-consuming for use in a busy ED. Although a shorter form with 5 items is validated for use internationally [26, 33], it was never investigated for the purposes of screening in a Nepali ED. We aimed to establish the diagnostic accuracy of the original GDS-5 from the GDS-15. Hoyl et al. [33] used clinical evaluation as the gold standard for depression, and revealed that the GDS-5 had a sensitivity of .97 and specificity of .85 for predicting depression. They reported the mean administration times for the 5- and 15-item GDS to be 9 and 2.7 min, respectively. In the extensive study done by Rinaldi et al. [26], the GDS-5 had a sensitivity of 0.94, a specificity of 0.81, and showed a significant agreement with the clinical diagnosis of depression (kappa = 0.74). In comparison, our study showed lower sensitivity of 0.75 with the original GDS-5 and a higher specificity of 0.96 with the acceptable kappa value of 0.73. However, Cronbach’s alpha was low (0.416). A new version of the 5-item GDS for our population was created selecting the 5 items from the GDS-15 with the highest corrected item-total correlation (items 1,3,4,7,14). The sensitivity thus improved to 0.90 and the Cronbach’s alpha increased to 0.623 with acceptable kappa value of 0.75 with GDS-15. This new version of GDS-5 is recommended for further use in the busy ED for screening purposes in our context.

### Limitations

Our population was a convenience sampling due to limited availability of the research assistant; thus, sampling bias cannot be excluded. Cases might have been missed if the research nurse was unavailable, most importantly during holidays and evenings. However, there were no consistent times of day or days when these absences occurred, thus these losses would not routinely represent specific subcategories of people in the emergency room, such as those who came early or late in the day. However, for these reasons, our conclusions relate solely to our sample. Another issue to be noted is that the patients with known depression under treatment and those who were delirious or sick to participate were excluded. Thus, the high-risk group for depression was excluded from the study which means that the actual prevalence of the disease may be higher than that found in our study. They do not reflect those referable to a prevalence study of elders with depressive symptoms who present in our ED. The information about the previous visits to the ED in our hospital or in any other hospital may be unreliable as it was self-reported by the patient or family based on recall since we don’t have a national registry to trace the information. In addition, the GDS-15 was used as the criterion standard instead of a formal psychiatric interview focusing on Diagnostic and Statistical Manual-IV (DSM-IV) criteria for the diagnosis of depression, therefore determination of bona fide diagnoses of depression could not be included during this phase of the study. Qualitative analysis of some variables (such as visits to local healers, issues with transportation, employment, etc) and for refusals, was not done during this phase, thus the specific impact of these issues on the degree of depression, or even all the factors associated with the choice to go to the emergency room and/or participate, could not be done.

## Conclusions

The overall rate of unrecognized depression was high among the geriatric patient in our ED sample. This, combined with a local prevalence study in community, indicates that depression is an urgent problem in Nepal. The results of this novel study-in a small, very busy ED with minimal privacy, in a low-resourced country with low literacy rate-cannot be compared exactly with studies done in highly resourced settings. However, it demonstrates that such studies are nonetheless feasible and valuable as important initial steps in case-finding for depression among elders in LMICs. As the population ages and resources become increasingly limited, the emergency care providers should receive adequate training and education in screening for geriatric depression. We recommend that all geriatric patients presenting in the ED in LMICs be routinely evaluated for unrecognized depression before discharge. Our hope is that our methodology for developing the revised, brief GDS-5 will be helpful to others in enabling more efficient use of time in the ED.

In subsequent work, we will develop guidelines for the identification of depression in geriatric ED patients utilizing the new proposed GDS-5 and explore the success of referrals to the specialist psychiatrist care. We hope that this work, as well as our subsequent efforts, will take advantage of these innovations to provide improved care for the distressingly high level of depression among the elderly in Kavre Province, and that these efforts may assist elders elsewhere in Nepal as well.

## Supplementary information

**Additional file 1.** The English and Nepali version of GDS-15.

## Data Availability

The Nepali and English version of GDS-15 is available as Additional file 1. Further dataset used and/or analyzed for this study are available from the corresponding author on reasonable request.

## Abbreviations

AUC: Area under curve
DH: Dhulikhel Hospital
CI: Confidence interval
COPD: Chronic obstructive pulmonary disease
CVD: Cardiovascular disease
DSM-IV: Diagnostic and Statistical Manual IV
DM: Diabetes mellitus
ED: Emergency Department
EM: Emergency Medicine
EP: Emergency physician
GCS: Glasgow coma scale
IQR: Interquartile range
LMICs: Low- and middle-income countries
MSK: Musculoskeletal
ROC: Receiving operating characteristic
SAGE: Study on Global AGEing and Adult Health
SD: Standard deviation
SPSS: Statistical Package for the Social Sciences
WHO: World Health Organization
YLD: Years lived with disability
