# Supplementary material for: Unrecognized geriatric depression in the emergency Department of a Teaching Hospital in Nepal: prevalence, contributing factors, and metric properties of 5 item geriatric depression scale in this population
Source: BMC Psychiatry. 2020 Nov 11;20:533. doi: 10.1186/s12888-020-02910-8 (PMC7656752; doi:10.1186/s12888-020-02910-8)
Supplement: Supplementary file 1 — Additional file 1. The English and Nepali version of GDS-15. [file 12888_2020_2910_MOESM1_ESM.pdf]

Geriatric Depression Scale 15

Choose the best answer for how the patient felt over the past week.

1. Patient ID

____

2. Are you basically satisfied with your life?/तपाई आफ्नो जीवनदेखि सामान्यतया सन्तुष्ट हुनुहुन्छ ? 1 point

Mark only one oval.

- Yes
- No

3. Have you dropped many of your activities and interests?/के तपाइले आफुलाई मनपर्ने कुरा (इच्छाहरु) र चाहनाहरु गर्न छाड्नुभएको छ ? 1 point

Mark only one oval.

- Yes
- No

4. Do you feel that your life is empty?/के तपाई आफ्नो जीवन शुन्य (रिक्त / काम नलाग्ने) भएको महसुस गर्नुहुन्छ ? 1 point

Mark only one oval.

- Yes
- No

5. Do you often get bored?/के तपाइलाई प्रायजसो दिक्क (अल्छी) लाग्छ ? 1 point

Mark only one oval.

- Yes
- No

6. Are you in good spirits most of the time?/के तपाई प्रायजसो हसिलो मुद्रा (अवस्था) मा रहनुहुन्छ ? 1 point

Mark only one oval.

- Yes
- No

7. Are you afraid that something bad is going to happen to you?/के तपाई कुनै नराम्रो कुरा हुन्छ कि भनेर पिर/चिन्ता मान्नुहुन्छ ? 1 point

Mark only one oval.

- Yes
- No

8. Do you feel happy most of the time?/के तपाई प्रायजसो खुशी भएको महसुस गर्नुहुन्छ ? 1 point

Mark only one oval.

- Yes
- No

9. Do you often feel helpless?/के तपाई प्रायजसो आफू असहाय भएको महसुस गर्नुहुन्छ ? 1 point

Mark only one oval.

- Yes
- No

10. Do you prefer to stay at home, rather than going out and doing new things?/ के तपाई घर बाहिर गएर कुनै नया काम गर्नुभन्दा प्रायजसो घरमै बस्न रुचाउनु हुन्छ ? 1 point

Mark only one oval.

- Yes
- No

11. Do you feel you have more problems with memory than most?/के तपाई आजकल अरुहरुको तुलनामा आफ्नो स्मरण (सम्झना) शक्तिमा समस्या भएको जस्तै महसुस गर्नुहुन्छ ? 1 point

Mark only one oval.

- Yes
- No

12. Do you think it is wonderful to be alive now?/के तपाई हालसम्म जीवित रहन पाएकोमा आनन्दित भएको ठान्नुहुन्छ ? 1 point

Mark only one oval.

- Yes
- No

13. Do you feel pretty worthless the way you are now?/के तपाई हालको समयमा आफु बेकार छु जस्तो लाग्छ? 1 point

Mark only one oval.

- Yes
- No

14. Do you feel full of energy?/के तपाई आफुलाई फुर्तिलो महसुस गर्नुहुन्छ ? 1 point

Mark only one oval.

- Yes
- No

15. Do you feel that your situation is hopeless?/के तपाई आफ्नो स्थितिप्रति (स्थिति देखेर) निराश भएको महसुस गर्नुहुन्छ ? 1 point

Mark only one oval.

- Yes
- No

16. Do you think that most people are better off than you are?/के तपाइलाई तपाइकोभन्दा अरुहरुको स्थिति राम्रो भएको जस्तो लाग्दछ ? 1 point

Mark only one oval.

- Yes
- No

This content is neither created nor endorsed by Google.

Google Forms
